# Letter from Liverpool

**Published:** 1865-04

**Authors:** 


LIVERPOOL, March 26th, 1865.

EDITOR MEDICAL EXAMINER:—

*Dear Sir:*—I have for a few days been staying in this growing city; and have improved the opportunity to investigate its sanitary regulations and hygienic condition.

In 1846, the borough of Liverpool procured the passage of an act for the improvement of its sewerage and drainage, and for its sanitary regulations. This, as amended in 1854, constitutes the basis of the general sanitary laws of the Kingdom.

The City of Liverpool is situated upon the north-east bank of the river Mersey. The elevation above tide-water varies, but in all parts is amply sufficient for drainage. Its population, including its dependencies, is about 450,000. The climate is more uniform than that of Chicago. The highest average temperature for the last eighteen years has been 62.6 degrees for the month of July; and the lowest 40 degrees for the month of

January. In 1864, the mean temperature has been below the average—the lowest being 16.9 degrees on the 6th of January, and the highest average, for any one day, was 66.8 degrees, on the 30th of August. These extremes are much less than in the region of the North American lakes. The winds from the west and south-west are mild, those from the east and south-east cold.

The sewerage of the city is directly into the river Mersey, in which the tides are very high, at some seasons of the year reaching thirty-three feet, and producing a current of more than six miles an hour, and yet the deposit of the sewerage in the bed of the river, along its banks, and at the bar, is a serious cause of complaint. The odors emanating from the stream, especially at low water, are identical with those so familiar to the olfactories of Chicagonians, while engineers think that the bar at the mouth of the river is materially augmented by the solid matter brought down from the city. The influence of this method of sewerage upon the health, and the commercial interests of the city, is being carefully investigated by men of the highest scientific abilities. If Liverpool suffers from this cause, with its river daily swept by the high tides and rapid currents, what must be the condition of Chicago with, in a very few years, an equal population?

I am under obligations to the Health Committee for the sanitary acts, and also for the report of the Medical Officer, for the year 1864. The sanitary acts have reference to sewerage, construction of dwellings, and especially lodging-houses, location and government of slaughter-houses, manufactures that influence the public health, and all other matters that have a bearing upon the hygienic condition of the city.

The report of Dr. FRENCH, the Medical Officer, is full of valuable information. Some of the facts which it contains may interest your readers. It appears that during the last three years the mortality has been steadily increasing. For the year 1864, it amounted to 36 deaths to every 1000 of its inhabitants. The increased percentage of mortality was due mainly to pulmonary and zymotic diseases, and especially to the latter, which caused 28.9 per cent. of all the deaths for the year.

Typhus fever, with typhoid, and infantile remittent, included in the report under the general head of typhus, seems to have been epidemic throughout the year. In 1860, the total number of cases under treatment in the city was 618; in 1864, the number reached 4157. The ratio of mortality to the number of cases treated, varies with the location and circumstances of the patients, from two per cent. in the better class of families, where, indeed, it seldom occurred, to twenty per cent. in the Toxteth fever wards. The report says, referring to the death registry of typhus:—"We find that though the weekly lists occasionally give fallacious indications of subsidence, yet the quarterly returns showed a steady increase, not only in the numbers of its victims, but in the extent and range of its fatal prevalence." In 1860, typhus produced 3 per cent. of all the deaths; in 1862, it accounted for 5.2 per cent.; in 1863, for 7.6 per cent., and in 1864, for 10.5 per cent. of all the deaths—thus showing a steady increase for the three years.

In discussing the causes of this epidemic, some important facts are brought to light. It appears that a large proportion of the poorer class of people find it difficult to obtain employment. Thus, in 1861, the number of public paupers was increased from something less than 13,000 to 15,000, and in 1862 to 16,000; while the number of persons relieved by charitable institutions was much greater. The Council of the District Provident Society relieved in 1861, 82,334 individuals, a large excess over previous years; and in 1863, the number reached 182,093. This seems enormous, but is based on official reports. And yet Englishmen have told me that there is no pauperism, or almost none, in England, that there is no destitution for the want of work. The statistics of Liverpool show, either that here, at least, labor is in excess, that bones, muscles, and nerves are more abundant than iron, wool, and cotton, or, that the nations of the world are less dependent than formerly on the manufactures of Great Britain.

The subject of overcrowding dwelling and lodging-houses, is also discussed. The smallest amount of air space sanctioned by the Health Committee of Liverpool is 300 cubic feet, with the means of rapid renewal. Practically, this amount is often reduced; whole families live and sleep in rooms where there is not on an average one-half of this space. In the registered lodging-houses, the regulations can be enforced, and but few cases of fever have been found in them. On the other hand, in the private dwelling-houses, the most fearful cases occurred, and almost always in overcrowded rooms. This overcrowding not only developed disease, but, in many cases, exhibited a very depraved social and moral sensibility. Cases are reported, where, in small rooms, without any furniture, men and women were found lying promiscuously on the bare floor, and one case I find reported, of two *young married couples* occupying the same room, with only one straw mattress for a bed. The sanitary act forbids the erection of houses of more than three stories, and a large number of houses front narrow streets, and have no back yards, being built up against other houses, with no back windows, even. These are called "up and down" houses, and amount to 18,000; nearly all of them are more or less overcrowded.

The accumulation of emigrants here, has been one cause of occasional overcrowding. The report mentions one peculiarity of the Irish and German emigration during the last year. Formerly, the inspectors found among them the average number of children and persons of advanced age, but in 1864, they are nearly all of them active young men and women, and amount in all to 125,445. In 1860, the total from this port was 83,774.

The report demonstrates that filth has been largely instrumental in producing and keeping up the epidemic. In fact, filth always accompanies destitution and is often produced by it. Drunkenness is also shown to have kept pace with the epidemic, and has no doubt acted as a cause. It is a sad comment upon human reason, that absolute starvation should be attended with this vice, which brings neither strength nor comfort, but such is the fact. The history of the epidemic, and of the people, during the last three years, points to destitution as its chief cause.

The conclusions of the reporter upon this subject, are in the following words:—

"1st. Great distress in the laborer's class, accompanied by an enormous increase of parochial pauperism, existed previously to the Winter of 1861 and Spring of 1862, when the typhus began to be in permanent excess.

"2d. The distress, and the prevailing fever, were both so simultaneously increased during 1862, as to indicate the parallelism of cause and effect.

"3d. The number of recipients of workhouse relief in 1863, though reduced by 186 out of 16,003 in 1862, showed that in spite of a partial revival of trade, the want was exceptionally great as compared with the ten years before 1861.

"4th. The transactions of the Central Relief Society, and the condition of the fever patients of the workhouse hospital in 1864, demonstrate that whatever might be the bettered circumstances in trade or commerce, the distress was not alleviated among the persons immediately above the rank of habitual paupers.

"5th. It was of necessity, and by reason, *firstly*, of want of employment, and, *secondly*, especially in 1864, by the losses sustained in their homes by sickness, that so many individuals of the working classes ceased to contribute their deposits in the "District Provident Society."*

"6th. The pressure which prevented the better-off from exercising a prudent saving, threw the more necessitous into abject want."

Such are the conclusions of the reporter, and yet I find that even during this period of destitution, the streets of Liverpool were full of the indications of suddenly acquired wealth. Fortunes of large amount were realized by speculations in cotton, while English ships, monopolizing the carrying trade of the seas, reaped rich harvests; and thus Liverpool and New York became competitors in the strife for *shoddy* notoriety.

During 1864, the deaths from small-pox were 482, of which 96 cases were reported to have been vaccinated. In 1863, the deaths were 100; in 1861, it was hardly found in the death

* This is a Savings Bank, and the number of depositors in 1864 was 21,822 less than during the year 1861.

registry. These facts show the close relation between this disease and typhus, so far as the general causes are concerned.

Diarrhœa was also more prevalent, especially during the months of July and August, of the year 1864, than during previous years. These months were remarkable for their extreme dryness, the amount of rain being less than one-half of that of the same months of 1861, while the mean temperature was somewhat greater. The deaths from diarrhœa, for the quarter ending September, 1861, was 222; for the corresponding quarter of 1864, 601.

Dr. French suggests whether the effect of rain in diminishing diarrhœa, may not in part be due to the fact that by it the streets are thoroughly washed, and the sewers flushed. In this way, a good rain storm may carry off more filth in a day, than all the scavengers in a week. He suggests the liberal use of the river water, through hydrants, upon the streets, and into the sewers. The query is pertinent to Chicago, as well as to Liverpool.

The mortality from scarlatina and measles, unlike that of small-pox, has been below the average during last year. Lung diseases have been, if not more frequent, at least more fatal, with the exception of tuberculosis. The increased fatality is attributed by Dr. French to the lower temperature of the year. He says:—"The popular fallacy, which assumes that cold weather is tonic, bracing, and healthy, or the old adage, which teaches that a mild winter makes a full churchyard, cannot have originated in cities, where man is enfeebled by the cares, the vices, and the sanitary evils of a highly artificial civilization." In regard to tuberculous diseases, it is remarked that, "the bills of mortality from this class of complaints are always light in very sickly seasons, or when zymotics are unusually fatal." No attempt is made to account for this fact, if fact it be.

